# Women’s knowledge, attitude, and practice regarding cervical precancerous lesions: a cross-sectional study in Beijing, China

**DOI:** 10.3389/fpubh.2024.1433718

**Published:** 2024-10-02

**Authors:** Lingyan Wang, Qianping Wang, Xia Zhou, Huan Liu

**Affiliations:** ^1^Department of Gynecology, The Eastern Area of Dongzhimen Hospital of Beijing University of Chinese Medicine, Beijing, China; ^2^Department of TCM Gynecology, China-Japan Friendship Hospital, Beijing, China; ^3^Department of Gynecology, Beijing Shijingshan District Hospital of Traditional Chinese Medicine, Beijing, China; ^4^Department of Gynecology, Beijing Xicheng Guangwai Hospital, Beijing, China

**Keywords:** knowledge, attitude, practice, women, cervical precancerous lesions, China, cross-sectional study

## Abstract

**Background:**

This study aimed to examine the knowledge, attitude, and practice (KAP) of women in Beijing regarding cervical precancerous lesions.

**Methods:**

This web-based, cross-sectional study included women at Dongzhimen Hospital of Beijing University of Chinese Medicine between March 13, 2024 and April 9, 2024. A self-administered questionnaire was developed to collect participants’ demographic information and KAP scores toward cervical precancerous lesions.

**Results:**

The study included 951 valid questionnaires, with a mean age of 40.0 years. The mean knowledge, attitude, and practice scores were 12.55 ± 6.23 (possible range: 0–26), 50.66 ± 7.48 (possible range: 13–65), and 26.13 ± 4.98 (possible range: 7–35), respectively. The knowledge score (OR = 1.071, 95%CI: 1.040–1.103, *p* < 0.001), currently married (OR = 0.548, 95%CI: 0.304–0.985, *p* = 0.045), and with a history of HPV infection (OR = 2.302, 95%CI: 1.062–4.990, *p* = 0.035) were independently associated with the attitude score. The knowledge score (OR = 1.155, 95%CI: 1.119–1.192, *p* < 0.001), monthly income >20,000 (OR = 2.793, 95%CI: 1.249–6.248, *p* = 0.012), a history of HPV infection (OR = 0.380, 95%CI: 0.222–0.650, *p* < 0.001), unknown HPV infection status (OR = 0.289, OR = 0.177–0.473, p < 0.001), vaccinated against HPV (OR = 1.752, 95%CI: 1.221–2.514, *p* = 0.002), giving birth to one child (OR = 1.991, 95%CI: 1.186–3.341, *p* = 0.009), and giving birth to two or more children (OR = 2.160, 95%CI: 1.240–3.763, *p* = 0.007) were independently associated with the practice score. The structured equation model showed that knowledge directly influenced attitude (*β* = 0.237, *p* = 0.004) and practice (*β* = 0.490, *p* = 0.010). Attitude directly influenced practice (*β* = 0.193, *p* = 0.009).

**Conclusion:**

This study revealed inadequate knowledge, moderate attitude, and practice toward cervical precancerous lesions among women in Beijing. Educational interventions should be developed to enhance knowledge in this regard.

## Background

Cervical cancer (CC) is a malignancy originating in the transformation zone of the cervix, most commonly in squamous cells (1). CC is the second most common cancer in women worldwide (estimated 661,021 new cases in 2022, or 3.3% of all cancers) and the third most common cause of cancer-related mortality in women (estimated 348,189 related deaths in 2022, or 3.6% of all cancer-related deaths) (49). Notably, CC is largely preventable. Over the last 30 years, the incidence and mortality of CC in high-income countries have decreased by more than 50%, a trend attributed to the implementation of formal screening programs (2, 3). CC is mainly prevented by screening for and treating cervical precancerous lesions (4). Cervical precancerous lesions are abnormal cells that can progress to CC without intervention and include cervical intraepithelial neoplasia grade 2–3 (CIN2-3) and adenocarcinoma *in situ* (AIS) (4).

Since CIN constitutes a continuum of progression, detecting and managing the lesions as soon as possible is conducive to improving prognosis (5–7). Although radical surgery can be necessary for advanced CC, early CC can be treated using fertility-sparing options (8, 9), such as laparoscopic-assisted vaginal trachelectomy. The collaboration of the women is essential for screening and managing cervical lesions. Indeed, sexually active women should undergo cervical screening regularly, request HPV vaccination, and follow their physicians’ advice (10, 11). Hence, proper knowledge and attitude of the women are prerequisites for the proper practice of CC prevention, including regarding precancerous lesions.

Knowledge, attitude, and practice (KAP) survey is a tool that provides quantitative and qualitative data about the gaps, misconceptions, and misunderstandings regarding a specific subject in a specific population (12, 13). Previous studies revealed highly variable KAP levels regarding cervical lesions and CC screening among different woman populations around the globe (14–20). A previous study examined the KAP toward CC screening among an ethnic minority in China (21), but no study examined the KAP toward cervical precancerous lesions in Beijing (China). In particular, the Beijing Municipal Health Commission issued the “Implementation Plan for Accelerating the Elimination of Cervical Cancer in Beijing (2023–2030)” in September 2023, stating that free cervical cancer screenings will progressively extend coverage to the entire permanent population of the whole city (22). Against this backdrop, there is an urgent need to investigate the current awareness among women in Beijing regarding cervical precancerous lesions.

Therefore, the objective of this study was to explore the KAP of Chinese women in Beijing regarding cervical precancerous lesions, with the aim of identifying critical areas where educational interventions are urgently required to improve awareness and preventive behaviors within the target population.

## Methods

### Study design and participants

This cross-sectional study included female inpatients, outpatients, and medical workers at the Dongzhimen Hospital of Beijing University of Chinese Medicine between March 13, 2024, and April 9, 2024. Women aged over 18 years, with clear consciousness, and capable of independently responding to the questionnaire were included in the study. No exclusion criteria were applied in this research. The research protocol was approved by the Ethics Committee of Dongzhimen Hospital Affiliated with the Beijing University of Chinese, under the approval number 2024DZMEC-093-02. All participants provided written informed consent before completing the questionnaire.

### Questionnaire

The design of the questionnaire was based on the 2021 WHO Guidelines: Screening and Treatment of Precancerous Lesions for Cervical Cancer Prevention (Second Edition) (23) and the related literature (6, 11, 24). Two gynecology experts, each with over 20 years of experience, rigorously reviewed the questionnaire items to ensure their accuracy and relevance, leading to the removal of any items deemed incorrect or inappropriate, thereby enhancing content validity. A total of 46 valid questionnaires were collected during the pre-survey, revealing a Cronbach’s *α* coefficient of 0.868, with values of 0.906, 0.700, and 0.761 for the knowledge, attitude, and practice sections, respectively, suggesting good internal consistency. During the pilot study, participants were encouraged to provide feedback on any items they found confusing or unclear, and no items were reported, thus confirming face validity. Finally, a confirmatory factor analysis was conducted, which demonstrated good model fit with the following indices: CFI (Comparative Fit Index) = 0.896, IFI (Incremental Fit Index) = 0.897, TLI (Tucker-Lewis Index) = 0.885, RMSEA (Root Mean Square Error of Approximation) = 0.060, and CMIN/DF (Chi-square value/degrees of freedom) = 4.285.

The final questionnaire was in Chinese and comprised four sections: (1) demographic information, including age, height, body weight, education level, HPV vaccination history, and sexual activity history, (2) knowledge dimension, (3) attitude dimension, and (4) practice dimension. The Body Mass Index (BMI) is calculated as the weight in kilograms divided by the square of the height in meters, with classifications of <18.5 kg/m^2^ as underweight, 18.5–23.9 kg/m^2^ as normal weight, 24–27.9 kg/m^2^ as overweight, and ≥ 28 kg/m^2^ as obesity (25). The knowledge (K) dimension included 13 questions, with scores ranging from 0 to 26, where 2 points were assigned for a “very familiar” response, 1 point for “heard about,” and 0 points for “unclear.” The knowledge dimension of this study encompassed a comprehensive understanding of cervical cancer, including its definition (items 1), risk factors (items 2–3), early detection and screening (items 4–7), treatment options (items 8–10), and HPV vaccination (items 11–13). The attitude (A) dimension consisted of 13 questions using a 5-point Likert scale, ranging from “strongly agree” (5 points) to “strongly disagree” (1 point), with total scores ranging from 13 to 65 points. The practice dimension included nine questions about practice frequency, rated from “never” to “always” and assigned values from 1 to 5. Items P8 and P9 did not show positive or negative attitude tendencies, and hence, a descriptive analysis of this question was performed, and the possible score range of practice dimension was 7–35 points.

### Questionnaire distribution and quality control

An online questionnaire was created using the Sojump website,^1^ and a QR code was generated for data collection via WeChat. The participants scanned the QR code to log in and complete the questionnaire. To ensure the quality and completeness of the questionnaire, all items were mandatory. If participants encountered any problem in answering, members of the research group were responsible for interpreting and solving the problem. After questionnaire collection, data quality checks were conducted, and questionnaires with logical errors or repeated pattern choices were considered invalid and excluded.

### Statistical analysis

The minimal sample size was estimated based on 10 times the number of demographic information and KAP items based on the sample size estimation methods for surveys (26). Hence, the minimal sample size was 550. When accounting for a 20% invalid questionnaire rate, the minimal sample size was 660.

SPSS 26.0 (IBM, Armonk, NY, United States) and AMOS 24.0 (IBM, Armonk, NY, USA) were used for the analysis. The continuous variables were described as means ± standard deviations and analyzed using Student’s t-test or ANOVA. The categorical variables were described as *n* (%) and analyzed using the chi-square test. Pearson correlation was used to analyze the correlation among the KAP scores. Univariable and multivariable logistic regression analyses were used to analyze the attitude and practice scores using 70% of the total score as the cutoff value. Univariable and multivariable logistic regression analyses were also used to analyze the factors influencing HPV vaccination. The variables with *p* < 0.05 in the univariable analyses were included in the multivariable analyses. A structural equation modeling (SEM) analysis was conducted to test the hypotheses that (H1) knowledge directly affects attitude, (H2) knowledge directly affects practice, and (H3) knowledge indirectly affects practice through attitude. Two-sided *p*-values <0.05 were considered statistically significant.

## Results

A total of 951 questionnaires were collected; 3 were missing informed consent, 1 participant reported <18 years of age, 13 had incomplete data or logical errors, and 19 where the participants chose “uncertain” for all knowledge-related questions. After the exclusions, 915 valid questionnaires were included for analysis. 18–44 age range (63.28%), maintained a normal BMI (50.60%), resided in urban areas (65.03%), were non-smokers (96.28%), currently married (80.33%), premenopausal (82.62%), not vaccinated against HPV (68.09%), and had no personal history of cervical lesions (77.49%), as outlined in Table 1.

**Table 1:** Characteristics and KAP scores of the participants.

	*n* (%)	Knowledge	*P*	Attitude	*P*	Practice	*P*
Total score (*N* = 915)		12.55 ± 6.23		50.66 ± 7.48		26.13 ± 4.98	
Age			0.001		<0.001		0.807
18–44 years	579 (63.28)	12.75 ± 6.12		51.78 ± 7.03		26.06 ± 5.14	
45–59 years	305 (33.33)	12.52 ± 6.31		48.50 ± 7.75		26.29 ± 4.70	
≥ 60 years	31 (3.39)	9.00 ± 6.59		51.10 ± 8.36		25.77 ± 4.72	
BMI			0.159		0.051		0.027
Underweight	45 (4.92)	12.40 ± 5.88		52.22 ± 7.21		25.13 ± 4.51	
Normal weight	463 (50.60)	12.94 ± 6.29		50.94 ± 7.17		26.52 ± 4.92	
Overweight	259 (28.31)	12.32 ± 6.28		49.54 ± 7.51		26.06 ± 4.92	
Obese	148 (16.17)	11.74 ± 5.99		51.27 ± 8.23		25.30 ± 5.33	
Residence			0.895		0.004		0.995
Urban	595 (65.03)	12.66 ± 6.26		51.23 ± 6.91		26.15 ± 5.02	
Rural	320 (34.97)	12.33 ± 6.17		49.61 ± 8.33		26.09 ± 4.93	
Education			<0.001		0.001		0.001
Junior high school and below	173 (18.91)	10.98 ± 5.98		49.06 ± 8.60		25.38 ± 4.97	
High school/ technical school	133 (14.54)	11.38 ± 6.20		49.31 ± 8.03		24.89 ± 5.05	
College	198 (21.64)	12.83 ± 6.20		51.37 ± 7.34		26.31 ± 5.10	
Bachelor’s degree	344 (37.60)	13.54 ± 6.13		51.67 ± 6.83		26.70 ± 4.84	
Master’s degree and above	67 (7.32)	12.97 ± 6.49		50.22 ± 5.63		27.06 ± 4.66	
Family monthly income (CNY)			0.004		0.231		0.002
< 2000	90 (9.84)	10.36 ± 6.43		49.82 ± 8.04		24.61 ± 4.77	
2000–5,000	269 (29.40)	12.29 ± 6.05		50.10 ± 8.28		25.70 ± 5.23	
5,000–10,000	268 (29.29)	13.22 ± 6.47		51.25 ± 6.95		26.34 ± 4.96	
10,000-20,000	196 (21.42)	12.93 ± 6.11		51.27 ± 7.08		26.61 ± 4.97	
> 20,000	92 (10.05)	12.62 ± 5.67		50.13 ± 6.59		27.24 ± 4.11	
Smoking status			0.141		0.025		0.595
Yes	34 (3.72)	11.35 ± 7.70		53.56 ± 6.72		25.76 ± 6.32	
No	881 (96.28)	12.59 ± 6.16		50.55 ± 7.48		26.14 ± 4.93	
Marital status			0.183		0.001		<0.001
Currently married	735 (80.33)	12.69 ± 6.17		50.30 ± 7.36		26.53 ± 4.82	
Unmarried or other	180 (19.67)	11.96 ± 6.44		52.15 ± 7.78		24.49 ± 5.29	
Menopausal status			0.027		0.001		0.850
Yes	159 (17.38)	11.54 ± 6.61		48.71 ± 7.94		26.06 ± 4.64	
No	756 (82.62)	12.76 ± 6.13		51.07 ± 7.32		26.14 ± 5.06	
HPV infection history			<0.001		<0.001		<0.001
Yes	90 (9.84)	15.70 ± 6.08		53.87 ± 6.17		26.28 ± 5.05	
No	712 (77.81)	12.52 ± 6.08		50.16 ± 7.54		26.58 ± 4.83	
Not checked, uncertain	113 (12.35)	10.18 ± 6.22		51.27 ± 7.39		23.16 ± 4.89	
HPV vaccination status			<0.001		<0.001		<0.001
Yes	292 (31.91)	13.96 ± 6.12		52.09 ± 6.97		27.58 ± 4.79	
No	623 (68.09)	11.88 ± 6.17		50.00 ± 7.61		25.45 ± 4.93	
Past cervical diseases			0.188		0.152		0.122
Yes	206 (22.51)	13.09 ± 6.22		51.26 ± 7.07		25.73 ± 4.82	
No	709 (77.49)	12.39 ± 6.23		50.49 ± 7.59		26.24 ± 5.03	
Family members of cervical cancer			0.351		0.263		0.618
Yes	10 (1.09)	14.80 ± 7.00		53.20 ± 6.96		25.80 ± 4.42	
No	905 (98.91)	12.52 ± 6.22		50.63 ± 7.48		26.13 ± 4.99	
Sexual activity history			0.214		0.417		<0.001
Yes	826 (90.27)	12.63 ± 6.17		50.60 ± 7.46		26.33 ± 4.90	
No	89 (9.73)	11.76 ± 6.72		51.20 ± 7.69		24.26 ± 5.35	
Contraceptive use			0.004		0.127		<0.001
Yes	733 (80.11)	12.84 ± 6.16		50.87 ± 7.30		26.54 ± 4.80	
No	182 (19.89)	11.36 ± 6.37		49.82 ± 8.10		24.46 ± 5.34	
Reproductive status			0.171		0.011		<0.001
Nulliparous	216 (23.61)	11.91 ± 6.18		52.00 ± 7.15		24.54 ± 5.09	
Gave birth to one child	393 (42.95)	12.71 ± 6.41		50.39 ± 7.31		26.56 ± 4.80	
Gave birth to two or more children	306 (33.44)	12.78 ± 6.01		50.06 ± 7.82		26.69 ± 4.92	
History of miscarriage			0.487		0.598		0.136
Yes	463 (50.60)	12.42 ± 6.32		50.54 ± 7.71		26.39 ± 4.82	
No	452 (49.40)	12.67 ± 6.14		50.79 ± 7.23		25.86 ± 5.14	

The mean knowledge score was 12.55 ± 6.23 (possible range: 0–26). Higher knowledge scores were observed in younger women (*p* = 0.001), with higher education (*p* < 0.001), with a higher income (*p* = 0.004), premenopausal (*p* = 0.027), with a history of HPV infection (p < 0.001), vaccinated against HPV (*p* < 0.001), and using contraceptives (*p* = 0.004) (Table 1). Among all the items, the item with the largest proportion choosing “very familiar” was K7 (30.16% very familiar; Free regular cervical cancer screening was organized annually in Beijing) and K12 (34.86% very familiar; There are three types of HPV vaccines available in China—bivalent, quadrivalent, and nonavalent—all of which are approved and can be received at the individual’s expense). On the contrary, the item with the largest proportion choosing “unclear” was K4 (36.28% unclear; Patients with cervical precancerous lesions generally do not exhibit obvious symptoms.) and K9 (43.39% unclear, Low-grade cervical precancerous lesions may naturally regress, requiring only regular follow-up without the need for treatment) (Supplementary Table S1).

The mean attitude score was 50.66 ± 7.48 (possible range: 13–65). Higher attitude scores were observed in the 18–44 age group (*p* < 0.001), urban residents (*p* = 0.004), with higher education (*p* = 0.001), smoking (*p* = 0.025), unmarried (*p* = 0.001), premenopausal (*p* = 0.001), with a history of HPV infection (*p* < 0.001), vaccinated against HPV (*p* < 0.001), and nulliparous (*p* = 0.011) (Table 1). Supplementary Table S2 shows the distribution of the responses to the attitude items.

The mean practice score was 26.13 ± 4.98 (possible range: 7–35). Higher practice scores were observed in women with normal BMI (*p* = 0.027), with higher education (*p* = 0.001), with higher income (*p* = 0.002), currently married (*p* < 0.001), without known status of HPV infection (*p* < 0.001), vaccinated against HPV (*p* < 0.001), history of sexual activity (*p* < 0.001), using contraceptives (*p* < 0.001), and with children (*p* < 0.001) (Table 1). Supplementary Table S3 shows the distribution of the responses to the practice items. Notably, 41.31% of participants indicated that they “always” or “often” undergo regular screening for cervical precancerous lesions. Besides, the most common means of obtaining information about cervical precancerous lesions were social media (41.31%), hospital lectures, and physicians (19.02%), while the predominant channels of obtaining information about HPV vaccination were social media (38.36%) and advice from relatives and friends (21.64%).

As shown in Table 2, the knowledge score was correlated to the attitude (*r* = 0.228, *p* < 0.001) and practice (*r* = 0.454, *p* < 0.001) scores. The attitude score was correlated to the practice score (*r* = 0.175, *p* < 0.001).

**Table 2:** Correlations among knowledge, attitude, and practice.

	Knowledge	Attitude	Practice
Knowledge	1		
Attitude	0.228 (*P* < 0.001)	1	
Practice	0.454 (*P* < 0.001)	0.175 (*P* < 0.001)	1

The results of multivariate regression revealed that knowledge score (OR = 1.071, 95%CI: 1.040–1.103, *p* < 0.001), marital status (OR = 0.548, 95%CI: 0.304–0.985, *p* = 0.045), and a history of HPV infection (OR = 2.302, 95%CI: 1.062–4.990, *p* = 0.035) were independently associated with the attitude score (Table 3). The knowledge score (OR = 1.155, 95%CI: 1.119–1.192, *p* < 0.001), monthly income >20,000 (OR = 2.793, 95%CI: 1.249–6.248, *p* = 0.012), a history of HPV infection (OR = 0.380, 95%CI: 0.222–0.650, p < 0.001), unknown HPV infection status (OR = 0.289, OR = 0.177–0.473, *p* < 0.001), HPV vaccination status (OR = 1.752, 95%CI: 1.221–2.514, *p* = 0.002), giving birth to one child (OR = 1.991, 95%CI: 1.186–3.341, *p* = 0.009), and giving birth to two or more children (OR = 2.160, 95%CI: 1.240–3.763, *p* = 0.007) were independently associated with the practice score (Table 4). The practice score (OR = 1.092, 95%CI: 1.052–1.133, *p* < 0.001) and age ≤ 44 years (OR = 3.681, 95%CI: 1.168–11.606, *p* = 0.026) were independently associated with receiving vaccination against HPV (Table 5).

**Table 3:** Univariate and multivariable logistic regression analysis of attitude.

	Univariable logistic regression		Multivariable logistic regression	
	OR (95%CI)	*P*	OR (95%CI)	*P*
Knowledge score	1.079 (1.050–1.109)	<0.001	1.071 (1.040–1.103)	<0.001
Age				
≤ 44 years	1.589 (0.691–3.651)	0.276		
45–59 years	0.672 (0.291–1.555)	0.353		
≥ 60 years	ref			
BMI				
< 18.5	1.498 (0.611–3.669)	0.377		
18.5–24	0.964 (0.616–1.510)	0.873		
24–28	0.690 (0.429–1.109)	0.125		
> 28	ref			
Residence				
Urban	1.925 (1.410–2.628)	<0.001	1.444 (0.972–2.146)	0.069
Rural	ref		ref	
Education				
Junior high school and below	ref		ref	
High school/ technical school	0.453 (0.229–0.896)	0.023	0.908 (0.539–1.531)	0.718
College	0.522 (0.257–1.058)	0.071	1.110 (0.652–1.888)	0.701
Bachelor’s degree	0.868 (0.434–1.736)	0.689	1.276 (0.741–2.198)	0.379
Master’s degree and above	1.187 (0.609–2.315)	0.615	1.102 (0.496–2.448)	0.812
Family monthly income				
< 2000	ref			
2000–5,000	0.774 (0.399–1.499)	0.447		
5,000–10,000	0.729 (0.423–1.258)	0.257		
10,000-20,000	1.305 (0.741–2.301)	0.357		
> 20,000	1.446 (0.791–2.641)	0.231		
Smoking status				
Yes	2.377 (0.828–6.823)	0.108		
No	ref			
Marital status				
Currently married	0.484 (0.310–0.757)	0.001	0.548 (0.304–0.985)	0.045
Unmarried or other	ref		ref	
Menopausal status				
Yes	0.512 (0.353–0.741)	<0.001	0.803 (0.520–1.241)	0.323
No	ref		ref	
HPV infection history				
Yes	3.468 (1.646–7.308)	0.001	2.302 (1.062–4.990)	0.035
No	ref		ref	
Not checked, uncertain	1.027 (0.649–1.626)	0.909	1.103 (0.673–1.808)	0.698
HPV vaccination status				
Yes	2.007 (1.399–2.879)	<0.001	1.458 (0.988–2.153)	0.057
No	ref		ref	
Past cervical diseases				
Yes	1.270 (0.869–1.856)	0.217		
No	ref			
Sexual activity history				
Yes	0.686 (0.390–1.206)	0.190		
No	ref			
Contraceptive use				
Yes	1.294 (0.895–1.870)	0.171		
No	ref			
Reproductive status				
Nulliparous	ref		ref	
Gave birth to one child	0.615 (0.400–0.945)	0.027	0.954 (0.543–1.677)	0.871
Gave birth to two or more children	0.487 (0.314–0.755)	0.001	0.858 (0.480–1.532)	0.604

**Table 4:** Univariate and multivariable logistic regression analysis of practice.

	Univariable logistic regression		Multivariable logistic regression	
	OR (95%CI)	*P*	OR (95%CI)	*P*
Knowledge score	1.168 (1.135–1.203)	<0.001	1.155 (1.119–1.192)	<0.001
Attitude score	1.034 (1.015–1.053)	<0.001	1.020 (0.998–1.042)	0.078
Age				
≤ 44 years	0.884 (0.416–1.881)	0.750		
45–59 years	1.018 (0.470–2.203)	0.964		
≥ 60 years	ref			
BMI				
< 18.5	1.014 (0.516–1.993)	0.967		
18.5–24	1.339 (0.918–1.952)	0.130		
24–28	1.323 (0.875–2.000)	0.184		
> 28	ref			
Residence				
Urban	1.002 (0.756–1.327)	0.989		
Rural	ref			
Education				
Junior high school and below	ref		ref	
High school/ technical school	0.836 (0.530–1.318)	0.440	0.760 (0.436–1.325)	0.333
College	1.278 (0.841–1.941)	0.251	1.051 (0.605–1.825)	0.860
Bachelor’s degree	1.454 (0.998–2.117)	0.051	0.894 (0.521–1.535)	0.685
Master’s degree and above	1.987 (1.070–3.689)	0.030	1.448 (0.645–3.253)	0.370
Family monthly income				
< 2000	ref		ref	
2000–5,000	1.339 (0.830–2.161)	0.232	1.242 (0.681–2.267)	0.480
5,000–10,000	1.882 (1.160–3.052)	0.010	1.729 (0.910–3.283)	0.094
10,000-20,000	2.015 (1.211–3.353)	0.007	1.788 (0.895–3.572)	0.100
> 20,000	3.182 (1.690–5.990)	<0.001	2.793 (1.249–6.248)	0.012
Smoking status				
Yes	0.579 (0.291–1.150)	0.118		
No	ref			
Marital status				
Currently married	2.173 (1.562–3.023)	<0.001	1.270 (0.759–2.126)	0.362
Unmarried or other	ref		ref	
Menopausal status				
Yes	1.071 (0.750–1.529)	0.707		
No	ref			
HPV infection history				
Yes	0.776 (0.493–1.222)	0.274	0.380 (0.222–0.650)	<0.001
No	ref		ref	
Not checked, uncertain	0.211 (0.138–0.325)	<0.001	0.289 (0.177–0.473)	<0.001
HPV vaccination status				
Yes	2.142 (1.576–2.911)	<0.001	1.752 (1.221–2.514)	0.002
No	ref		ref	
Past cervical diseases				
Yes	0.821 (0.598–1.127)	0.223		
No	ref			
Sexual activity history				
Yes	0.886 (0.248–3.161)	0.852		
No	ref			
Contraceptive use				
Yes	2.252 (1.448–3.502)	<0.001	1.259 (0.832–1.905)	0.276
No	ref		ref	
Reproductive status				
Nulliparous	ref		ref	
Gave birth to one child	2.129 (1.517–2.989)	<0.001	1.991 (1.186–3.341)	0.009
Gave birth to two or more children	2.356 (1.644–3.376)	<0.001	2.160 (1.240–3.763)	0.007

**Table 5:** Univariate and multivariable logistic regression analysis of vaccination against HPV.

	Univariable logistic regression		Multivariable logistic regression	
	OR (95%CI)	*P*	OR (95%CI)	*P*
Knowledge score	1.055 (1.031–1.079)	<0.001	1.021 (0.993–1.050)	0.150
Attitude score	1.039 (1.019–1.060)	<0.001	1.011 (0.988–1.033)	0.355
Practice score	1.094 (1.062–1.128)	<0.001	1.092 (1.052–1.133)	<0.001
Age				
≤ 44 years	4.916 (1.698–14.232)	0.003	3.681 (1.168–11.606)	0.026
45–59 years	1.138 (0.380–3.410)	0.818	0.947 (0.303–2.961)	0.926
≥ 60 years	ref		ref	
BMI				
< 18.5	1.683 (0.839–3.372)	0.142		
18.5–24	1.358 (0.906–2.037)	0.138		
24–28	0.935 (0.596–1.467)	0.769		
> 28	ref			
Residence				
Urban	1.475 (1.093–1.991)	0.011	0.780 (0.518–1.175)	0.235
Rural	ref		ref	
Education				
Junior high school and below	ref		ref	
High school/ technical school	0.751 (0.409–1.379)	0.356	0.634 (0.317–1.271)	0.199
College	1.845 (1.135–2.997)	0.013	0.907 (0.479–1.716)	0.763
Bachelor’s degree	3.439 (2.226–5.311)	<0.001	1.657 (0.881–3.117)	0.117
Master’s degree and above	2.525 (1.353–4.712)	0.004	0.952 (0.421–2.153)	0.907
Family monthly income				
< 2000	ref		ref	
2000–5,000	1.239 (0.696–2.207)	0.466	0.591 (0.293–1.194)	0.143
5,000–10,000	1.736 (0.984–3.063)	0.057	0.656 (0.314–1.368)	0.261
10,000-20,000	2.688 (1.504–4.802)	0.001	0.933 (0.432–2.015)	0.860
> 20,000	2.750 (1.430–5.287)	0.002	0.910 (0.396–2.093)	0.825
Smoking status				
Yes	1.518 (0.756–3.050)	0.241	1.202 (0.693–2.085)	0.513
No	ref		ref	
Marital status				
Currently married	0.701 (0.499–0.984)	0.040		
Unmarried or other	ref			
Sexual activity history				
Yes	0.511 (0.328–0.795)	0.003	0.578 (0.309–1.082)	0.087
No	ref		ref	
Contraceptive use				
Yes	0.760 (0.541–1.068)	0.114		
No	ref			
Reproductive status				
Nulliparous	ref		ref	
Gave birth to one child	0.551 (0.389–0.779)	0.001	0.797 (0.465–1.366)	0.409
Gave birth to two or more children	0.527 (0.365–0.761)	0.001	0.777 (0.441–0.368)	0.381

**Figure 1:**
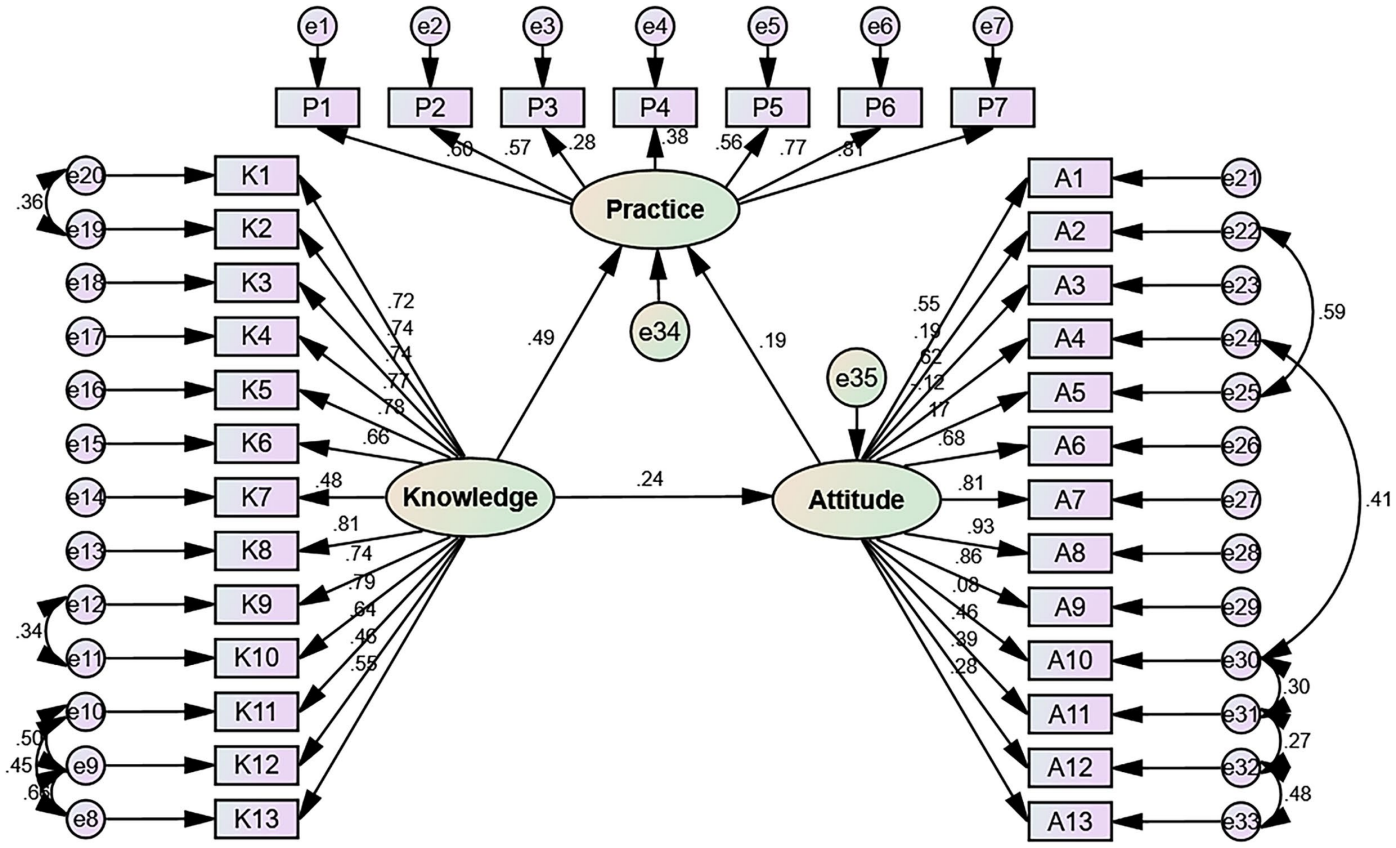
Structural equation model showing the associations among KAP scores. The direction of causality is indicated by single-headed arrows. The standardized path coefficients are presented alongside the arrows.

The SEM showed that knowledge directly influenced attitude (*β* = 0.237, *p* = 0.004) and practice (*β* = 0.490, *p* = 0.010). Attitude directly influenced practice (β = 0.193, *p* = 0.009). And knowledge indirectly influenced practice (*β* = 0.046, *p* = 0.006) (Table 6, Figure 1). Supplementary Table S4 shows that all fit indexes were good.

**Table 6:** SEM analysis.

Model paths	Standardized direct effects	P	95%CI	Standardized indirect effects	*P*	95%CI
Knowledge→Attitude	0.237	0.004	0.165–0.313			
Knowledge→Practice	0.490	0.010	0.417–0.553			
Attitude→Practice	0.193	0.009	0.116–0.265			
Knowledge→Practice				0.046	0.006	0.026–0.075

## Discussion

The results of this cross-sectional study revealed women in Beijing displayed poor knowledge, moderate attitude, and moderate practice toward cervical precancerous lesions. The current rate of vaccination against HPV is low. Educational interventions should be designed to improve knowledge of cervical precancerous lesions and HPV vaccination rates.

The responsibility for being screened for cervical lesions belongs to each woman, but proper knowledge of the advantages and risks of being screened or not must be well-understood to cultivate the proper attitudes that would lead to optimal practice. Previous studies generally reported relatively poor to moderate KAP toward cervical lesions in different countries. Indeed, a study in Ethiopia reported that 61% of the participants had heard of CC, and 72% were willing to be screened, but only 2% did (14). Similar results were reported in Uganda (16), India (17, 20), and Peru (18). In another study from Ethiopia, in a study population with a 27% rate of cervical precancerous lesions, 63 and 67% of the women had good knowledge and favorable attitudes, respectively (15). On the other hand, an Italian study reported good knowledge about the Pap test, CC, and HPV infection (19). A study on the Uyghur population in China showed that women had a poor knowledge of CC, and only 6% had heard of the HPV vaccine (21). Similarly, poor knowledge of CC prevention was also observed in Shenzhen (27) and Guizhou (28). The present study was performed in the general population of women in Beijing and not specifically in minorities, but the knowledge of CC and its prevention would nevertheless require improvements. Of course, the level of economic development and education the government provides can influence the women’s KAP. The present study reported poor knowledge but moderate attitude and practice toward cervical precancerous lesions among women in Beijing. Still, the standard deviations on the mean scores were high, indicating high variability among the participants. This disparity may stem from women’s inclination to heed advice from the local community or healthcare professionals, yet there is a lack of channels or interest in deeply understanding the underlying reasons behind these recommendations. The low knowledge scores identified in this study may also originate from traditional beliefs and misconceptions about HPV infection. Indeed, many Chinese women are influenced by traditional views prevalent in the East Asian culture and tend to associate cervical lesions and HPV infection with immorality, sexual shame, and sexually transmitted diseases (29, 30). This association often leads to reluctance among these women to learn more about this condition. In addition, a significant proportion of postmenopausal women and rural women hold the erroneous belief that menopause or the absence of sexual activity precludes the risk of developing cervical lesions. This misconception leads to the refusal to acquire relevant knowledge, consequently impacting their attitudes and practices toward the disease. It is recommended that efforts to disseminate knowledge about the progression of precancerous lesions be expanded within communities, health check-up centers, and medical institutions, alongside strengthened follow-up management for high-risk groups.

The present study suggests a complex interplay of factors influencing attitudes and practices regarding cervical precancerous lesions. Indeed, the results indicated that marriage was associated with a decreased concern regarding cervical precancerous lesions, possibly due to misconceptions about sexual risk factors (31). Indeed, it is true that the risk of cervical lesions increases with the number of sexual partners, but it is crucial to acknowledge that having a single sexual partner can also be associated with an increased risk of cervical lesions if the partner carries HPV (31–33). A history of HPV infection correlated with a more positive outlook, likely due to heightened awareness of medical explanations (15). Indeed, such women are more susceptible to having received detailed explanations about HPV infections and precancerous cervical lesions. Having an unchecked HPV status and having a history of HPV infection was also associated with poor practice. Having children was also associated with a better practice toward cervical lesions. It could be related to the higher risk of cervical lesions with parity and a closer follow-up by gynecologists (15). A high income was associated with more proactive practice toward cervical lesion screening, possibly related to better access to healthcare resources (15). This study found an association between lower educational levels and unsatisfactory KAP regarding cervical precancerous lesions, aligning with findings from prior research (34, 35). Given that women with lower socioeconomic status are at higher risk and represent the most affected demographic for cervical cancer (36–38), emphasizing screening promotion for these groups is crucial. This discrepancy requires the development of more customized health communication strategies that cater to the different backgrounds and needs of various demographic groups.

HPV vaccination is an important preventive measure against cervical lesions and CC (39). In the present study, the rate of HPV vaccination was relatively low, at 32%. In China, HPV vaccines are presently purchased privately, and the pilot program for HPV vaccine immunization among girls of eligible age in Beijing is scheduled to commence in 2023 (22). Therefore, the women must decide to be vaccinated. Being ≤44 years old was independently associated with being vaccinated against HPV, probably mainly because the approved age for HPV vaccines is under 45 years old (40). Besides, it is the period of higher sexual activity. In addition, HPV vaccines and their importance are relatively novel (39), and it is more likely that younger women were targeted by their physicians for advice on the vaccine. It is supported by a study that showed that age > 41 years was associated with a more unfavorable attitude toward HPV vaccination in China (28). A previous study in China showed that mothers of daughters were more willing to be vaccinated themselves and have their daughters vaccinated (27), but it was not observed in the present study, possibly because the sex of the participants’ offspring was not collected. Furthermore, novel technologies such as mRNA testing are being made available for the rapid and effective screening of HPV (41). Fertility-sparing options are increasingly available (9, 42), but considering the risk of preterm birth in women with cervical precancerous lesions treated with conization (43), the prevention of HPV infection with vaccination makes more sense.

The SEM analysis revealed that knowledge positively influenced attitude and practice and that attitude positively influenced practice. Hence, improving knowledge should also improve the attitude and practice of the women toward cervical precancerous lesions. The results showed poor knowledge about the nature of the lesions, the role of HPV, the risk factors for cervical lesions, symptoms, cervical testing methods, CC screening, treatments, the possibility of CIN regression, and the continuum from CIN to CC. Hence, future educational interventions should target those points in particular. Educational programs on the progression of precancerous lesions in communities, health check-up centers, and medical institutions should be performed, and the follow-up management of high-risk groups should be strengthened. A study in China showed that exposing young women to information about HPV-related diseases increased their intention of getting vaccinated (44).

This study had several limitations. The study was performed at a single hospital, limiting the patients to a given geographical area and generalizability of the results. In addition, the participants were selected based on predefined criteria, possibly introducing bias. The study was cross-sectional, preventing the analysis of causality, for which longitudinal studies are necessary. Nevertheless, an SEM analysis was performed to estimate causality among KAP dimensions, but SEM analyses are statistical surrogates based on predefined hypotheses, and the results must be taken cautiously (45, 46). The questionnaire was designed by the investigators according to local practice, culture, and policies, limiting the exportability of the questionnaire to other centers or geographical areas and the generalizability of the results. All KAP studies are at risk of social desirability bias, in which the participants can answer what they know they should think or do instead of what they are actually doing (47, 48). Nevertheless, the questionnaire was self-reported, possibly introducing bias due to understanding the questions and response choices. In addition, the questionnaire was web-based, introducing a selection bias due to the access to technology. This study focused solely on cervical precancerous lesions without accounting for broader factors influencing women’s health behaviors. Finally, although qualitative analyses would have enriched the results and conclusion, they were not performed.

Future research could focus on several key areas to address the identified limitations and unanswered questions. First, longitudinal studies would be valuable for establishing causal relationships between KAP related to cervical precancerous lesions and HPV vaccination. Additionally, research could explore the impact of targeted educational interventions on improving KAP scores, particularly to address the significant variability observed in this study, with a focus on increasing HPV vaccination rates among young, sexually active women. Furthermore, qualitative research could provide deeper insights into the underlying reasons for knowledge gaps and attitudes, thereby refining public health strategies.

## Conclusion

In conclusion, women in Beijing displayed poor knowledge and moderate attitude and practice toward cervical precancerous lesions. This underscores the urgent need for comprehensive educational strategies specifically tailored to the identified needs and challenges of the target population, aiming to enhance awareness and promote preventive behaviors among women in Beijing to reduce the incidence of cervical precancerous conditions.

## Data Availability

The original contributions presented in the study are included in the article/Supplementary material, further inquiries can be directed to the corresponding author.

## Footnotes

^1^ https://www.wjx.cn/
