# Supplementary material for: Women’s knowledge, attitude, and practice regarding cervical precancerous lesions: a cross-sectional study in Beijing, China
Source: Front Public Health. 2024 Oct 2;12:1433718. doi: 10.3389/fpubh.2024.1433718 (PMC11480777; doi:10.3389/fpubh.2024.1433718)
Supplement: Supplementary file 1 [file Data_Sheet_1.docx]

**Table S1. Knowledge dimension, n (%)**

|  | Very familiar | Heard about | Unclear |
| --- | --- | --- | --- |
| 1. Cervical precancerous lesions refer to abnormalities in the cervical epithelium with the potential for malignant transformation, and their long-term presence may lead to the development of cervical cancer. | 161 (17.6) | 544 (59.45) | 210 (22.95) |
| 1. The majority of cervical precancerous lesions are closely associated with persistent infection of high-risk human papillomavirus (HPV). | 171 (18.69) | 525 (57.38) | 219 (23.93) |
| 1. Risk factors for the occurrence of cervical precancerous lesions include early sexual activity, multiple sexual partners, multiparity, smoking, long-term oral contraceptive use, malnutrition, immunodeficiency, lack of awareness regarding health, and reluctance to undergo cervical cancer screening. | 161 (17.6) | 519 (56.72) | 235 (25.68) |
| 1. Patients with cervical precancerous lesions generally may not exhibit obvious symptoms. | 126 (13.77) | 457 (49.95) | 332 (36.28) |
| 1. Common screening methods for cervical precancerous lesions include cervical cytology examination, HPV testing, and colposcopy combined with histopathological examination. | 210 (22.95) | 493 (53.88) | 212 (23.17) |
| 1. Women aged 25 and above should commence cervical disease screening. | 219 (23.93) | 538 (58.8) | 158 (17.27) |
| 1. Free regular cervical cancer screening was organized annually in Beijing. | 276 (30.16) | 407 (44.48) | 232 (25.36) |
| 1. The choice of treatment for cervical precancerous lesions depends on the patient’s condition and the grade of the lesion. | 154 (16.83) | 467 (51.04) | 294 (32.13) |
| 1. Low-grade cervical precancerous lesions may naturally regress, requiring only regular follow-up without the need for treatment. | 117 (12.79) | 401 (43.83) | 397 (43.39) |
| 1. High-grade cervical precancerous lesions can progress to cervical cancer, necessitating surgery or ablative treatment based on the patient’s condition. | 137 (14.97) | 459 (50.16) | 319 (34.86) |
| 1. Vaccination against HPV and adopting a healthy lifestyle can prevent cervical precancerous lesions. | 249 (27.21) | 545 (59.56) | 121 (13.22) |
| 1. There are three types of HPV vaccines available in China—bivalent, quadrivalent, and nonavalent—all of which are approved and can be received at the individual’s expense. | 319 (34.86) | 490 (53.55) | 106 (11.58) |
| 1. The eligible age for HPV vaccination is 9-45 years, focusing on girls 9-14. | 263 (28.74) | 508 (55.52) | 144 (15.74) |

**Table S2. Attitude** **dimension, n (%)**

|  | Strongly agree | Agree | Neutral | Disagree | Strongly disagree |
| --- | --- | --- | --- | --- | --- |
| 1. I believe that screening and treatment for cervical precancerous lesions are crucial for women’s health. | 730 (79.78) | 142 (15.52) | 35 (3.83) | 3 (0.33) | 5 (0.55) |
| 1. I am concerned that I may have cervical precancerous lesions. | 212 (23.17) | 190 (20.77) | 333 (36.39) | 72 (7.87) | 108 (11.8) |
| 1. I am willing to undergo screening and treatment for cervical precancerous lesions to prevent the occurrence of cervical cancer. | 590 (64.48) | 211 (23.06) | 98 (10.71) | 2 (0.22) | 14 (1.53) |
| 1. I avoid going to the hospital for examination due to the fear of having cervical cancer. | 93 (10.16) | 101 (11.04) | 169 (18.47) | 164 (17.92) | 388 (42.4) |
| 1. I worry about the possibility of contracting HPV. | 167 (18.25) | 168 (18.36) | 297 (32.46) | 112 (12.24) | 171 (18.69) |
| 1. I consider HPV vaccination to be necessary. | 526 (57.49) | 226 (24.7) | 148 (16.17) | 6 (0.66) | 9 (0.98) |
| 1. I believe that screening for cervical precancerous lesions can reduce the risk of women developing cervical cancer. | 596 (65.14) | 215 (23.5) | 89 (9.73) | 6 (0.66) | 9 (0.98) |
| 1. I think screening and treatment for cervical precancerous lesions should be part of routine health check-ups for women. | 626 (68.42) | 192 (20.98) | 82 (8.96) | 7 (0.77) | 8 (0.87) |
| 1. I believe that screening and treatment for cervical precancerous lesions should receive attention and importance from society. | 667 (72.9) | 165 (18.03) | 75 (8.2) | 2 (0.22) | 6 (0.66) |
| 1. I am concerned about facing judgment from others due to HPV infection or cervical diseases. | 208 (22.73) | 165 (18.03) | 235 (25.68) | 136 (14.86) | 171 (18.69) |
| 1. I am worried that HPV infection and cervical precancerous lesions may progress to cervical cancer. | 375 (40.98) | 267 (29.18) | 211 (23.06) | 32 (3.5) | 30 (3.28) |
| 1. I believe that cervical precancerous lesions can impact sexual life. | 307 (33.55) | 260 (28.42) | 289 (31.58) | 36 (3.93) | 23 (2.51) |
| 1. I believe that cervical precancerous lesions can affect marital relationships. | 281 (30.71) | 235 (25.68) | 286 (31.26) | 66 (7.21) | 47 (5.14) |

**Table S3. Practice dimension, n (%)**

|  | Always | Often | Sometimes | Occasionally | Never |
| --- | --- | --- | --- | --- | --- |
| 1. I undergo regular screening for cervical precancerous lesions. | 171 (18.69) | 207 (22.62) | 270 (29.51) | 170 (18.58) | 97 (10.6) |
| 2. I pay attention to nutritional intake in my daily life. | 231 (25.25) | 322 (35.19) | 267 (29.18) | 79 (8.63) | 16 (1.75) |
| 3. I practice hygiene and use condoms in my sexual activities. | 353 (38.58) | 247 (26.99) | 168 (18.36) | 92 (10.05) | 55 (6.01) |
| 4. I maintain good perineal hygiene. | 553 (60.44) | 321 (35.08) | 36 (3.93) | 2 (0.22) | 3 (0.33) |
| 5. I regularly engage in physical exercise to stay active. | 245 (26.78) | 237 (25.9) | 273 (29.84) | 146 (15.96) | 14 (1.53) |
| 6. I encourage myself and female family members to get vaccinated against HPV. | 255 (27.87) | 221 (24.15) | 222 (24.26) | 157 (17.16) | 60 (6.56) |
| 7. I encourage female friends and family members around me to undergo screening and treatment for cervical precancerous lesions. | 305 (33.33) | 233 (25.46) | 224 (24.48) | 120 (13.11) | 33 (3.61) |
| 8. Where do you primarily acquire knowledge about cervical precancerous lesions: |  |  |  |  |  |
| Medical books and materials | 155 (16.94) |  |  |  |  |
| Hospital lectures and doctor’s education | 174 (19.02) |  |  |  |  |
| New media (WeChat, Weibo, etc.) | 378 (41.31) |  |  |  |  |
| Multimedia (TV and other news media) | 117 (12.79) |  |  |  |  |
| Relatives and friends | 91 (9.95) |  |  |  |  |
| 9. Where do you primarily acquire information about the HPV vaccine: |  |  |  |  |  |
| Recommendations from relatives and friends | 198 (21.64) |  |  |  |  |
| Medical books and materials | 129 (14.1) |  |  |  |  |
| Hospital lectures and doctor’s education | 153 (16.72) |  |  |  |  |
| New media (WeChat, Weibo, etc.) | 351 (38.36) |  |  |  |  |
| Multimedia (TV and other news media) | 84 (9.18) |  |  |  |  |

**Table S4. Goodness of fit of SEM**

| Model fit index | Reference | Measured results |
| --- | --- | --- |
| CMIN/DF | 1-3 excellent, 3-5 good | 4.938 |
| RMSEA | <0.08 good | 0.066 |
| IFI | >0.8 good | 0.875 |
| TLI | >0.8 good | 0.862 |
| CFI | >0.8 good | 0.874 |

CMIN/DF, Chi-Square divided by Degrees of Freedom; RMSEA, Root Mean Square Error of Approximation; IFI, Incremental Fit Index; TLI, Tucker-Lewis Index; CFI, Comparative Fit Index.
